# Supplementary material for: Characterization of SARS-CoV-2 intrahost genetic evolution in vaccinated and non-vaccinated patients from the Kenyan population
Source: J Virol. 2025 May 6;99(6):e00482-25. doi: 10.1128/jvi.00482-25 (PMC12172480; doi:10.1128/jvi.00482-25)
Supplement: Supplemental figures — Figures S1 to S11. [file jvi.00482-25-s0001.pdf]

Supplementary Fig. 1: Top 30 mutations in non-vaccinated and vaccinated patients in Kenya.

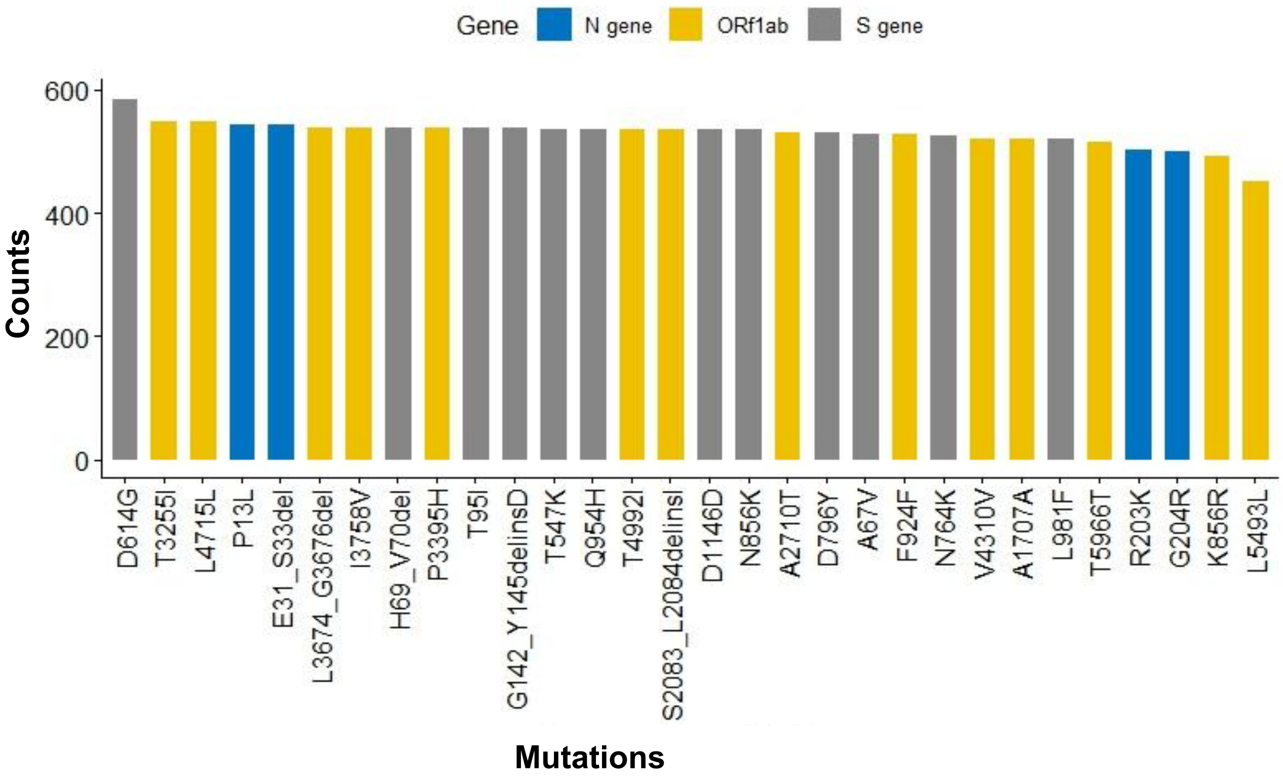

Supplementary Fig. 1: Shows the top 30 mutations in non-vaccinated and vaccinated patients in Kenya. Mutations were mostly found on the ORF1a/b, S, and N gene. Mutations in blue are found in the N genes, those in yellow are found in the ORF 1a/b, and those in grey are found in the S gene.

Supplementary Fig. 2: Recombination events per patient in non-vaccinated and vaccinated patients in Kenya.

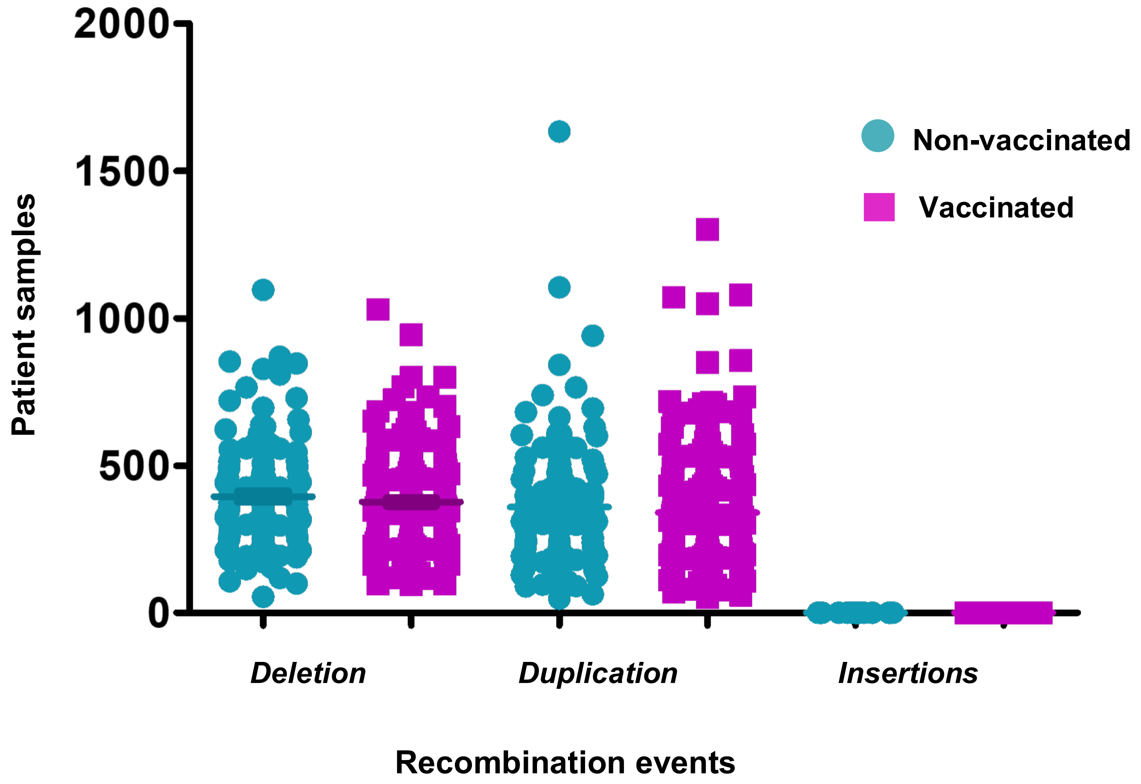

Supplementary Fig. 2: Shows the number of deletion, duplications, and insertion events per patient in non-vaccinated and vaccinated patients in Kenya. Green circles represent recombination events found in non-vaccinated patients and purple boxes represent those found in vaccinated patients.

Supplementary Fig. 3: Top recombination events between non-vaccinated and vaccinated individuals.

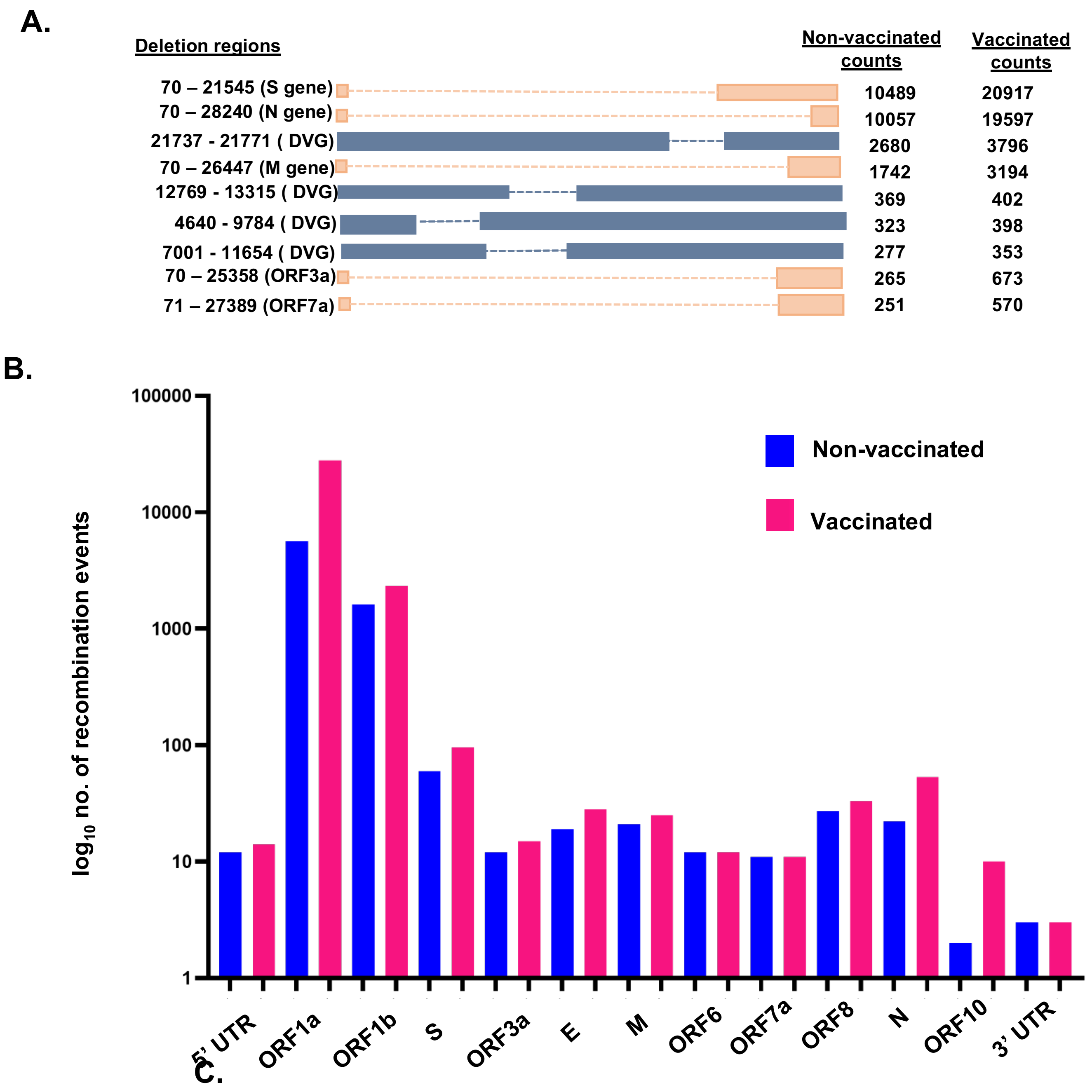

Supplementary Fig. 3: Top recombination events between non-vaccinated and vaccinated individuals. A. Top recombinant RNA species between vaccinated and non-vaccinated individuals and the counts. B & C. Recombination events based on major genome positions between vaccinated and non-vaccinated individuals.

Supplementary Fig. 4: JFreq (junction frequency) quantification of recombinant RNA species between sex (male and female), in the cohort of vaccinated and non-vaccinated individuals.

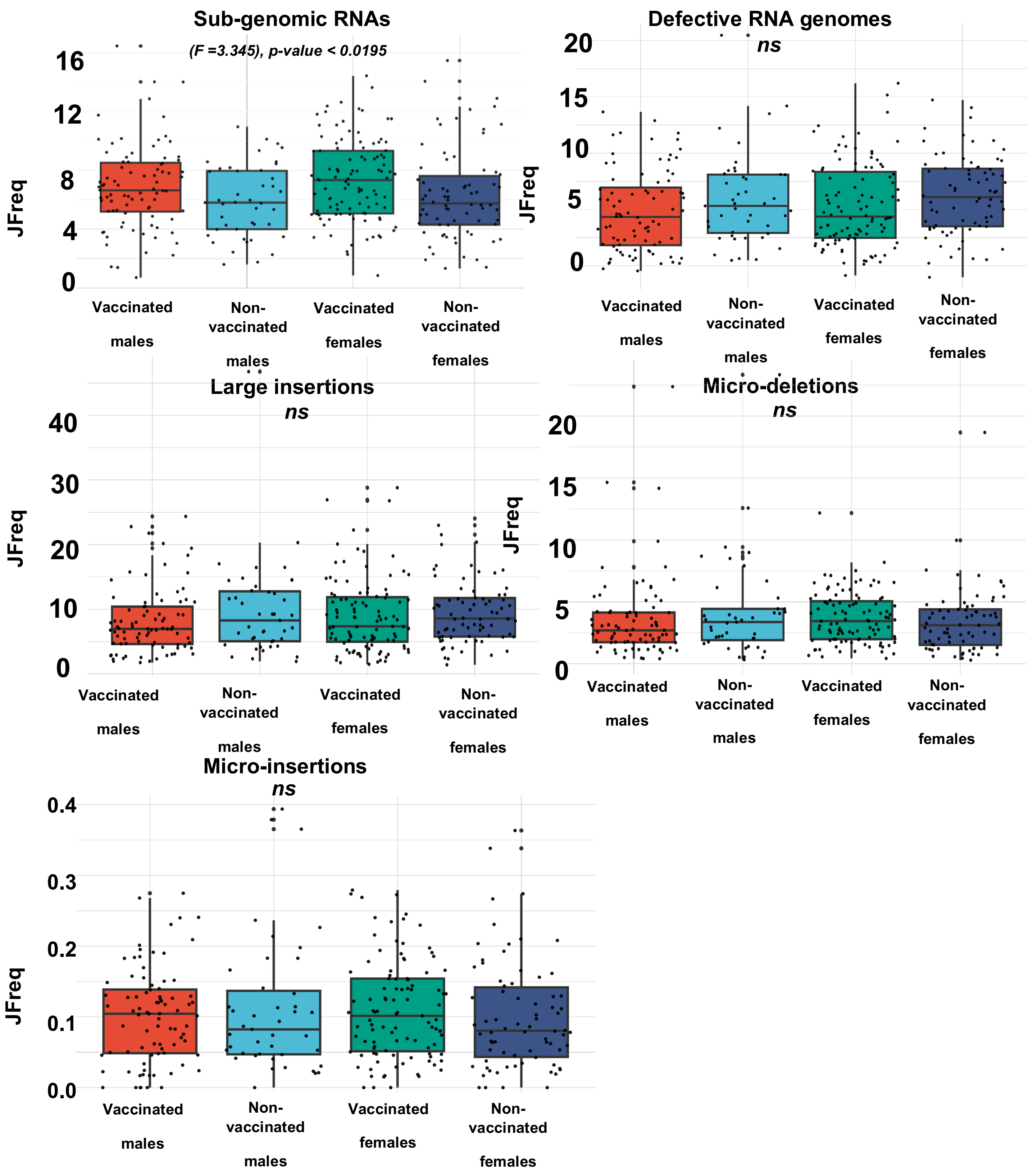

Supplementary Fig. 4: The boxplots represent the JFreq (junction frequency) quantification of the recombination RNA species between sex (male and female), in the cohort of vaccinated and non-vaccinated individuals. Statistical significance was determined using one way ANOVA and Tukey multiple comparison tests.

Supplementary Fig. 5: JFreq (junction frequency) quantification of the recombination RNA species between age groups (1-30, 31-50, above 50), in the cohort of vaccinated and non-vaccinated individuals.

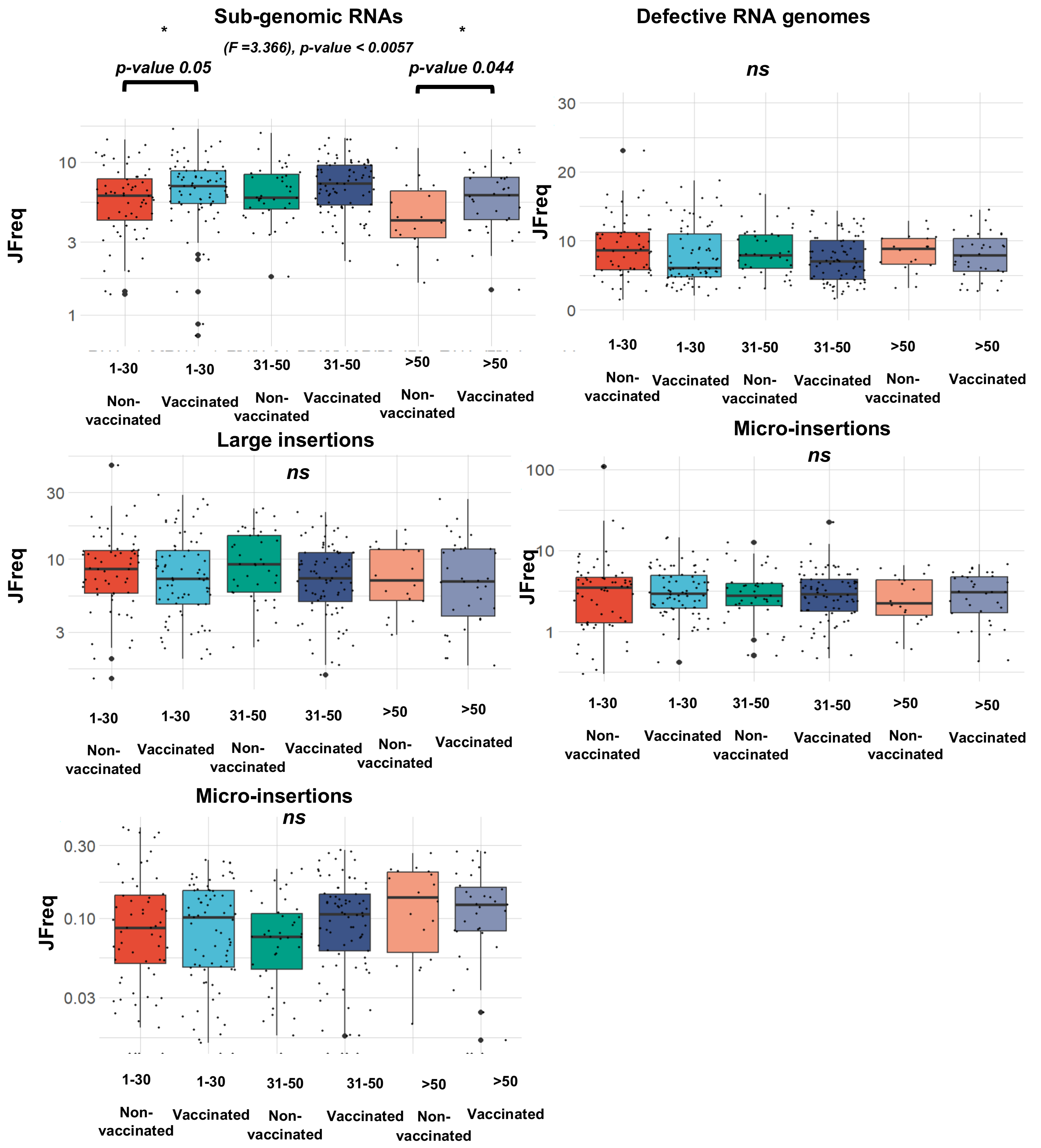

Supplementary Fig. 5: The boxplots represent the JFreq (junction frequency) quantification of the recombination RNA species between age groups (1-30, 31-50, above 50) in the cohort of vaccinated and non-vaccinated individuals. Statistical significance was determined using one way ANOVA and Tukey multiple comparison tests.

Supplementary Fig. 6: JFreq (junction frequency) quantification of sgmRNAs between complete, not complete vaccine dosage, in the cohort of vaccinated individuals.

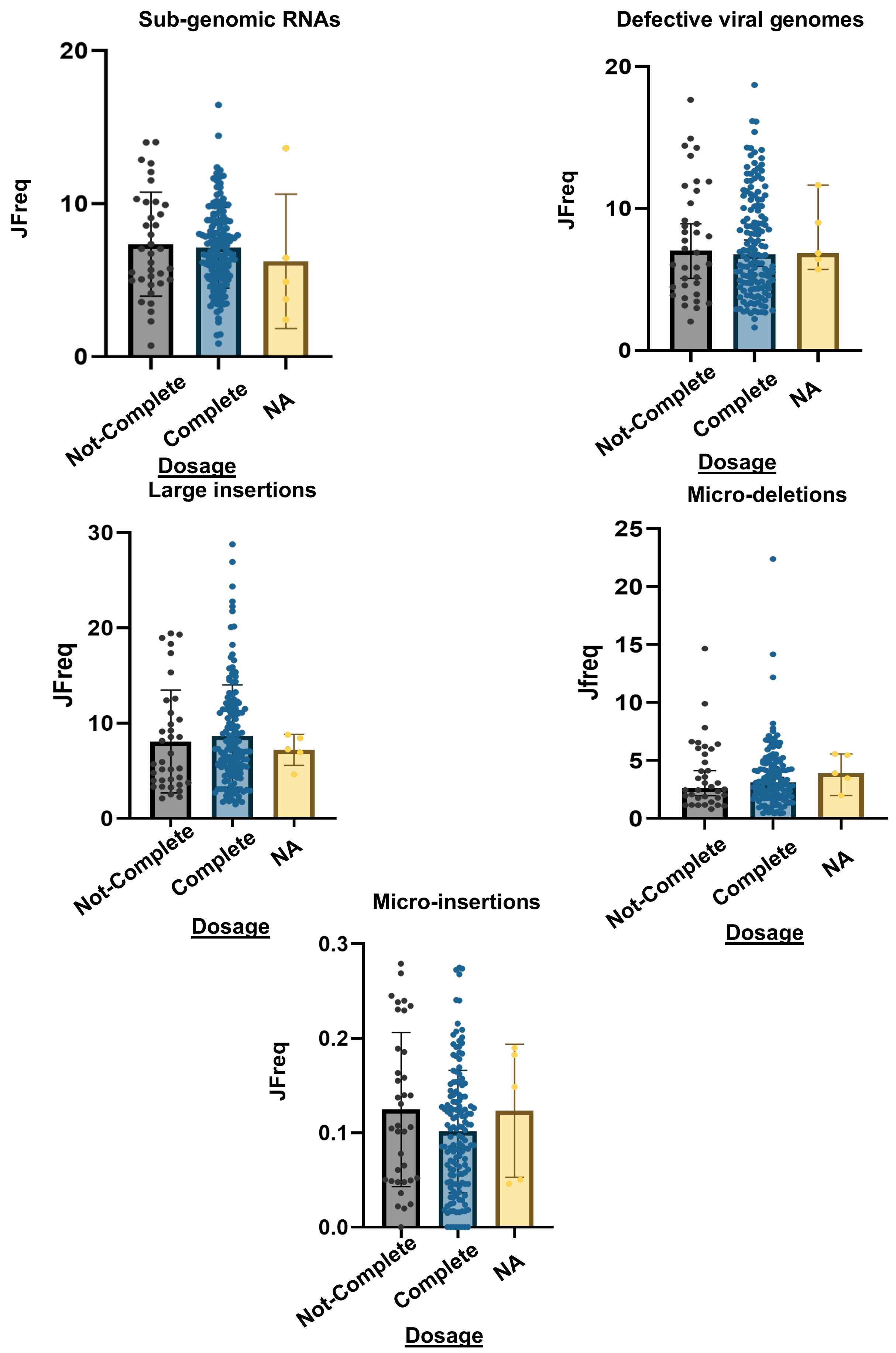

Supplementary Fig. 6: The boxplots represent the JFreq (junction frequency) quantification of the types of sgmRNAs between vaccine dosage , in the cohort of vaccinated individuals.

Supplementary Fig.7: JFreq (junction frequency) quantification of sgmRNAs between sex (male and female), in the cohort of vaccinated and non-vaccinated individuals.

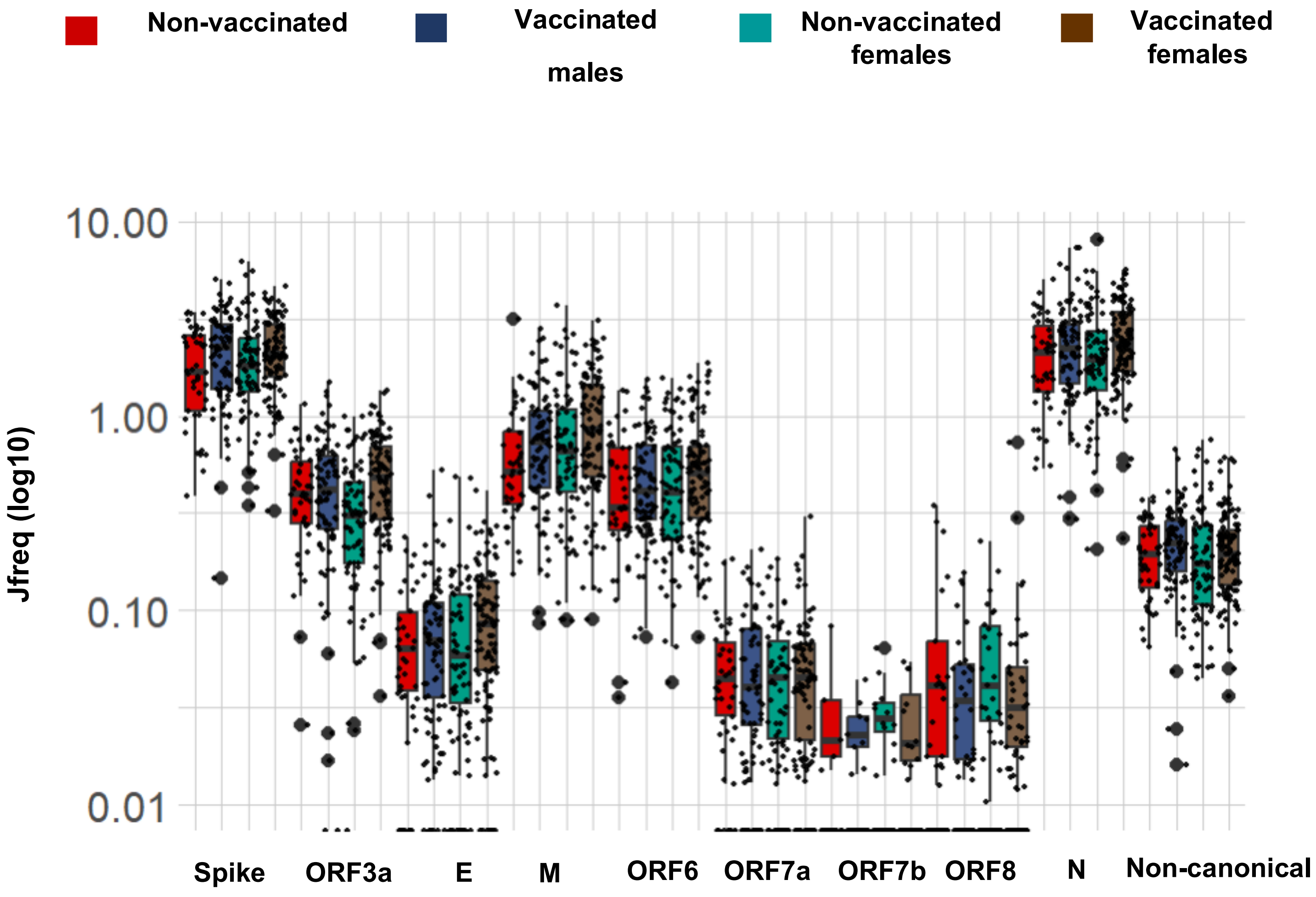

Supplementary Fig. 7: The boxplots represent the JFreq (junction frequency) quantification of the types of sgmRNAs between different sexes (male and female), in the cohort of vaccinated and non-vaccinated individuals.

Supplementary Fig. 8: JFreq (junction frequency) quantification of sgmRNAs between age groups (1-30, 31-50, above 50), in the cohort of vaccinated and non-vaccinated individuals.

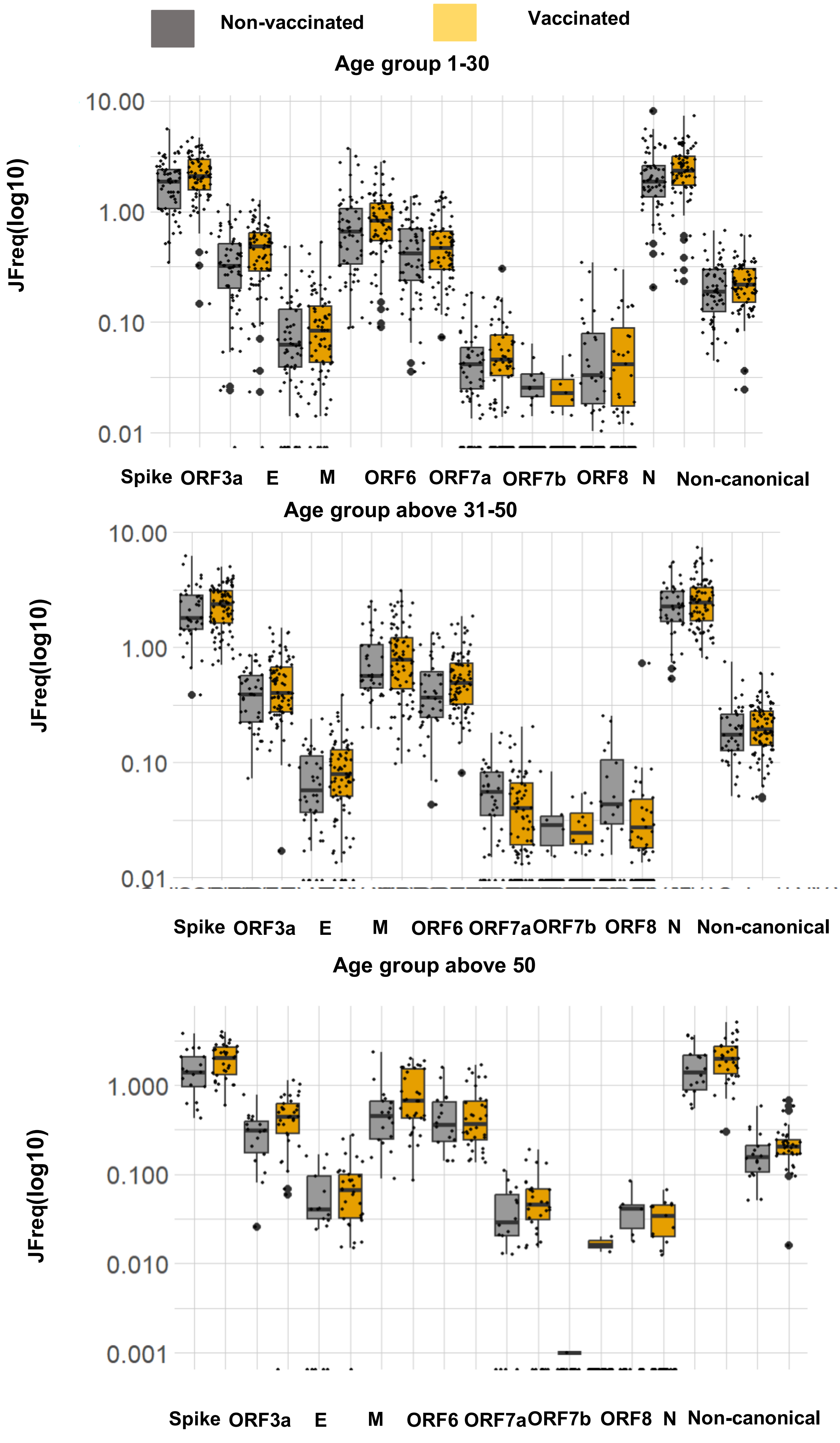

Supplementary Fig. 8: The boxplots represent the JFreq (junction frequency) quantification of the types of sgmRNAs between age groups (1-30, 31-50, above 50) , in the cohort of vaccinated and non-vaccinated individuals.

<u>Supplementary Fig. 9: Box plot of genome coverage between waves and interwaves of SARS-CoV-2 from sequences in a Kenyan population.</u>

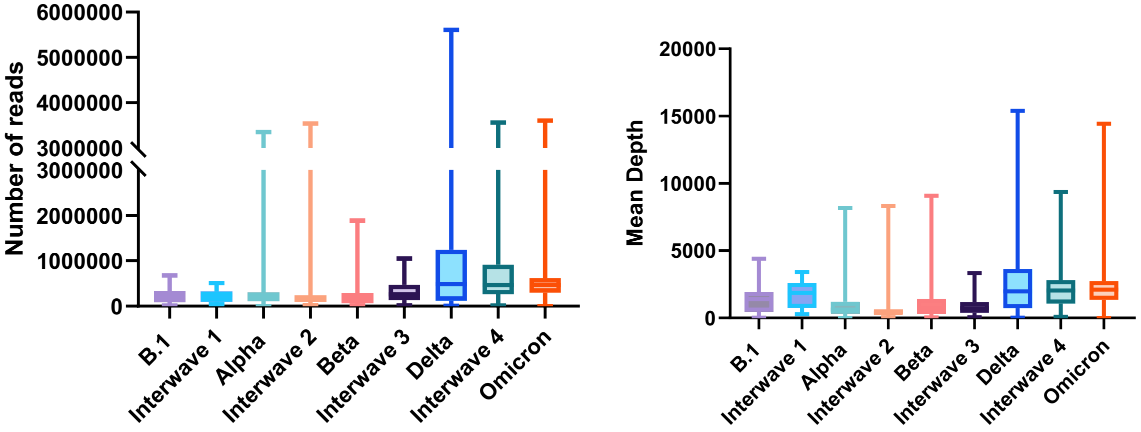

Supplementary Fig. 9: The boxplots represent the frequency of the number of reads and mean depth between all waves and interwaves.

<u>Supplementary Fig. 10 : ViReMa identifies recombination events between and during the peak of SARS-CoV-2 transmission waves.</u>

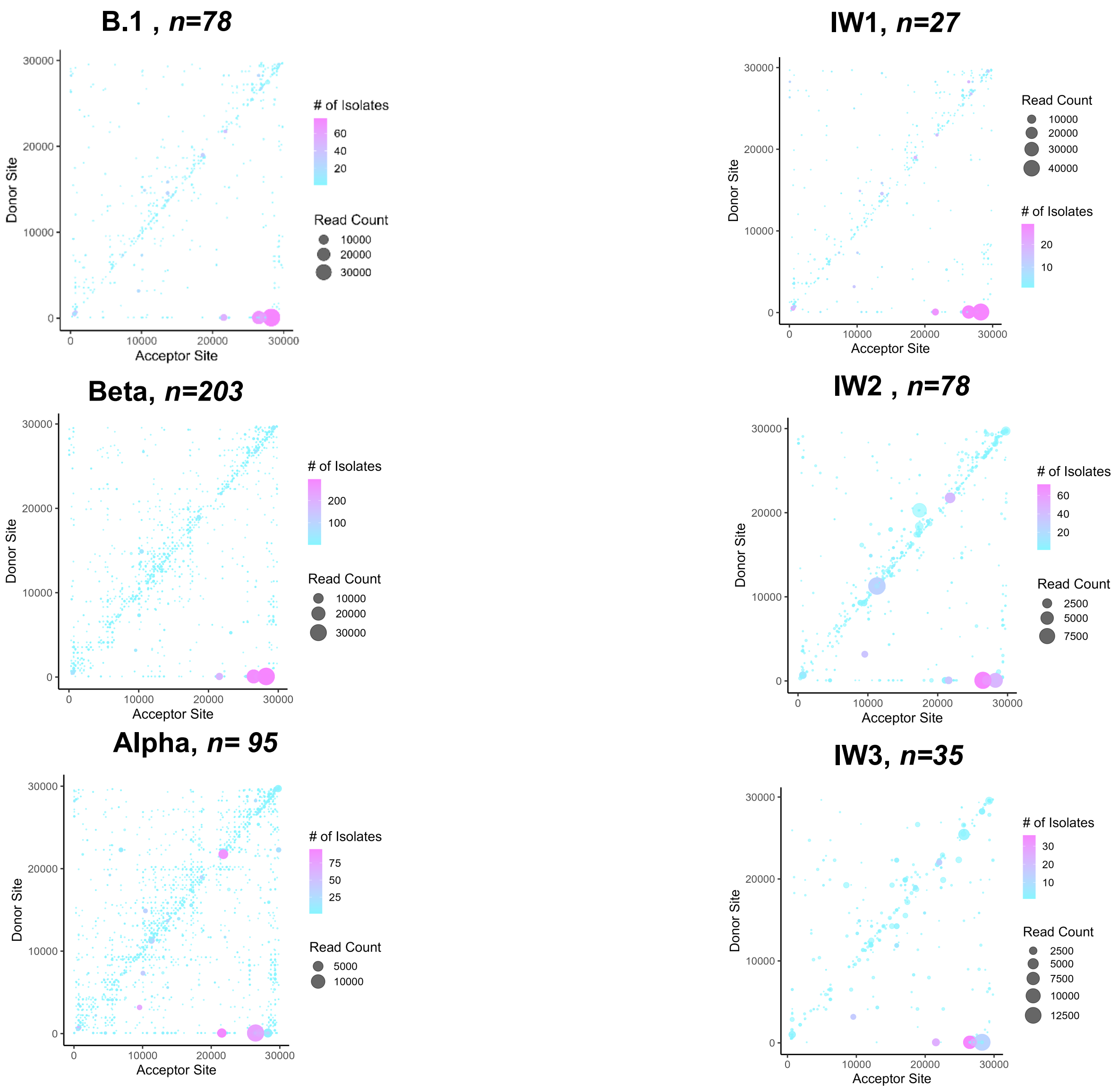

Supplementary Fig. 10: ViReMa scatter plots of SARS-CoV-2 recombination events and hotspots over B.1, interwave 1, Beta, interwave 2, Alpha, and interwave 3. The gradient in the scatter plot legend represents the number of patient samples containing a recombination event. The darker shaded circles in the scatter plot represent events that occur in multiple patient samples, while the circle size corresponds to the count of the reads of a recombination event.

Supplementary Fig. 11: Unique mutations in the ORF 1a/b, N, and S gene of non-vaccinated and vaccinated patients in Kenya.

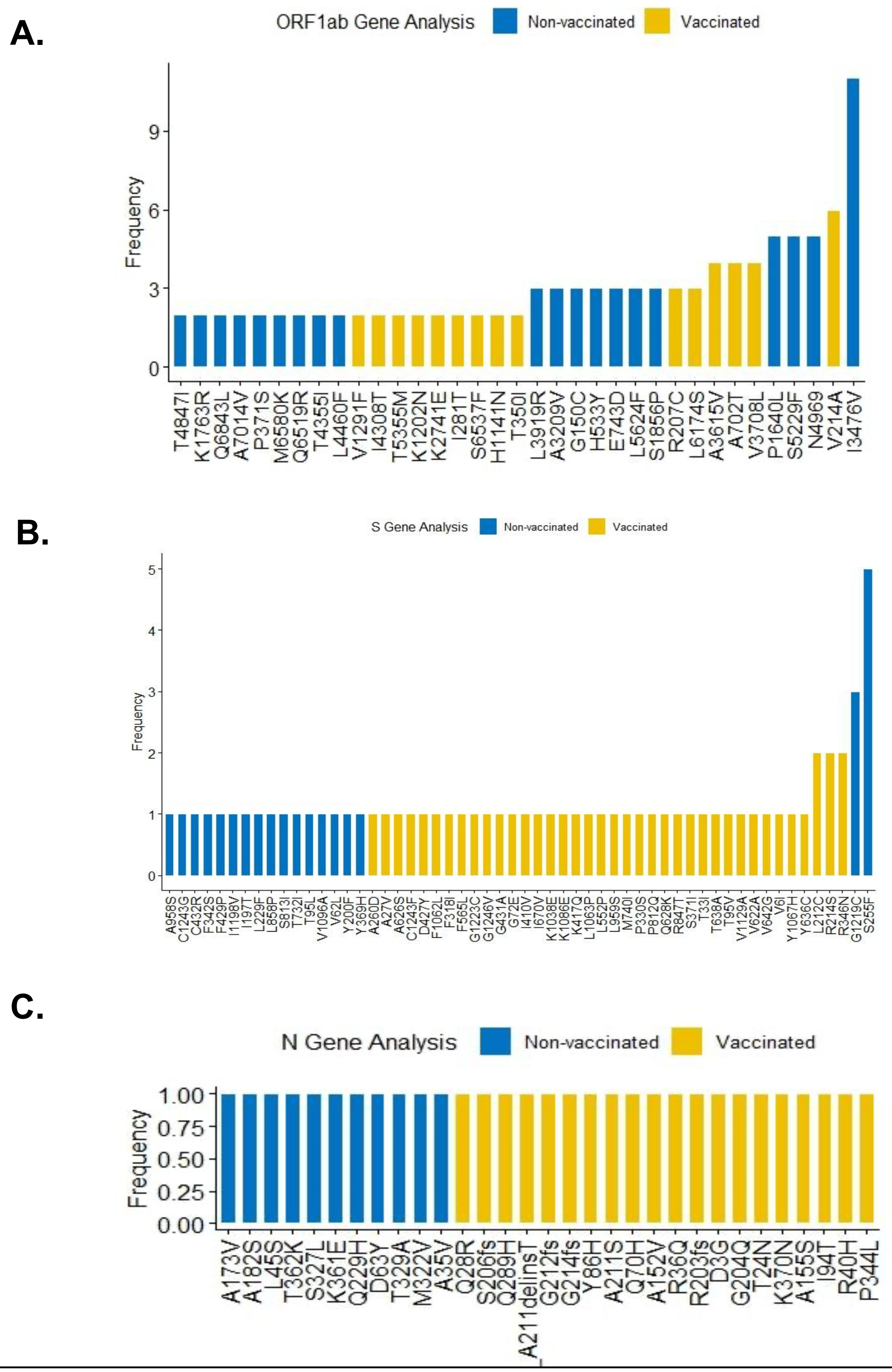

Supplementary Fig. 11: Unique mutations in non-vaccinated and vaccinated patients in Kenya. Mutations in blue represent those found in non-vaccinated patients and those in yellow represent those in vaccinated patients. A. Shows unique mutations in the ORF1 a/b. B. Shows unique mutations in the S genes C. Shows unique mutations in the N gene.
